# How to Evaluate Disability

**DOI:** 10.3389/fpsyt.2013.00054

**Published:** 2013-06-14

**Authors:** Zohaib Sohail, Rahn Kennedy Bailey, William D. Richie

**Affiliations:** ^1^Meharry Medical College, Sayreville, NJ, USA; ^2^Department of Psychiatry, Meharry Medical College, Nashville, TN, USA; ^3^Residency Training Program, Psychiatry Externship Program, Division of Forensic Psychiatry, Meharry Medical College, Nashville, TN, USA

**Keywords:** disability, impairment, psychiatry residency, independent psychiatric examination, AMA guidelines

## Abstract

A disability assessment for non-therapeutic reasons is the most common evaluation requested of treating psychiatrists. Mental disorders affect approximately 20 percent of Americans each year. People who are unable to work need some financial assistance. As part of the system, it’s our goal to assist them in this process. When a disability claim is filed, psychiatrists take into account the individual’s impairments and disabilities. A psychiatrist’s evaluation of disability involves knowledge and experience. There are many ethics related challenges, especially when performing disability evaluation of their own patients. Disability training should therefore be part of residency curriculum for training of psychiatry residents.

Psychiatrists are often unaware of the process of disability evaluation, as psychiatric residencies mostly do not train residents how to conduct them. This area of need prompts the necessity to outline guidelines that could assist psychiatrist in performing an accurate and valuable psychiatric disability evaluation.

According to different laws and insurance companies, Disability is a legal concept with more than one definition. It is similar to a competency but quite different from Impairment.

The most recommended *American Medical Association Guides to the Evaluation of Permanent Impairment Guides* (AMA’s), Fifth Edition (Andersson and Cocchiarella, 2008) defines Disability as an “Activity limitation and/or participation restriction in an individual with a health condition, disorder, or disease” (Andersson and Cocchiarella, 2008, p. 5) and Impairment as “a significant deviation, loss or loss of use of any body structure or body function in an individual with a health condition, disorder or disease” (Andersson and Cocchiarella, 2008, p. 5).

An impairment “results from anatomical, physiological, or psychological abnormalities which can be shown by medically acceptable clinical and laboratory diagnostic techniques” (United States Social Security Administration Office of Disability Programs, 2005). World Health Organization (WHO) defines Impairment as “problems in body function or structure such as a significant deviation or loss” (World Health Organization, 2002, p. 10). There are different classifications of impairment used which include the World Health Organization’s *International Classification of Functioning*, *Disability*, *and Health* (ICF) World Health Organization (WHO) (2001); Social Security Administration regulations C.F.R. pt. 404 (2005); DSM-IV-TR Global Assessment of Functioning (GAF) Scale (American Psychiatric Association, 2000).

Disability evaluation is often referred as an independent psychiatric examination that can be requested by employer, government institutions, insurance agencies, or either party in litigation. The purpose of such reports is to help them decide their course of action such as providing healthcare benefits or arranging damages and workplace accommodations.

Currently there are no standardized tests to evaluate an applicant’s mental health that could directly determine disability. Rating scales are sometimes helpful in quantifying impairment although not recommended. DSM GAF Scale (American Psychiatric Association, 2000) is a standard diagnostic assessment most commonly applied.

The AMA *Guides* (Andersson and Cocchiarella, 2008) advices physicians to maintain confidentiality while evaluating disability cases. It’s difficult routinely because of third-party evaluations, but a psychiatrist should try his level best in doing so. A consent form for release of pertaining relevant information (C.F.R. §164.508 (b)(4)(iii), 2007) according to The Health Insurance Portability and Accountability Act (HIPAA) (1996), should therefore be included as part of the records. Though there exists a limited doctor patient relationship (Weinstock and Garrick, 1995; Weinstock and Gold, 2004; Baum, 2005) in such evaluations, problem arises when a psychiatrist attempt to plays the dual role, as a treatment provider and that of an evaluator. This can lead to conflict of ethics. The American Academy of Psychiatry and the Laws (AAPL’s) guideline, therefore suggests that the primary treating psychiatrist avoid performing evaluations for legal purposes of their own patients (American Academy of Psychiatry and the Law, 2005).

There are many types of disability evaluations including social security, worker compensation, private disability insurance, American with Disabilities Act (ADA), fitness for duty, and return to work. All of them have some similarities and differences, which are summarized in Table 1 (Lisa et al., 2008).

**Table 1:** **Categories of disability evaluations**.

Column 1	Definition of disability provided	Causation relevant	Degree of impairment relevant	Partial or total disability	Litigation possible
SSDI	Yes	No	No	No	Yes
Workers compensation	No	Yes	Yes	Yes	Yes
Private disability insurance	Varies	No	Varies	Varies	Yes
American with disabilities act	Yes	No	Yes	N/A	N/A
Fitness for duty	No	No	Yes	N/A	Yes
Return to work	No	No	Yes	N/A	Yes

Although a psychiatrist should be aware of all types of disability evaluations individually, our aim is to provide general overview of pertinent common factors involved in performing any evaluation.

There are certain procedures that need to be taken into consideration in order to conduct a comprehensive psychiatric disability evaluation (see Table 2). A written informed consent stating that evaluation is not for treatment purpose but diagnosing level of impairment or disability should be signed before beginning a standard Psychiatric Examination. A psychiatrist should be aware of the time frame required to file a disability case along with the details of medical and legal events in evaluees life.

**Table 2:** **Summarizing a typical evaluation report**.

1.	Identifying information
2.	Source of referral and reason for referral
3.	Relevant legal standards and criteria
4.	Informed consent/statement of non-confidentiality
5.	Dates and durations of examinations
6.	Sources of information: third-party information including records reviewed, collaterals sources interviewed
7.	Relevant background information
	(a) Family history
	(b) Personal history
	(c) Education history
	(d) Employment history
	(e) Religious history
	(f) Military history
	(g) Sexual, marital, and relationship history
	(h) Medical history
	(i) Drug and alcohol history
	(j) Legal history (juvenile and adult crimes and civil matters)
	(k) Psychiatric history
8.	Relevant physical examination, imaging studies, and laboratory tests
9.	Psychological testing and assessment instruments administered
10.	Current mental status examination
11.	Competency examination data
12.	Clinical conclusions and diagnoses
13.	Medico legal conclusions including expert opinion competency if formulated
14.	Opinion on restorability and commitability
15.	Formulation and basis for the expert opinion(s)

It is necessary to clarify the referral source. Collateral information is gathered such as employment records, educational documents, substance abuse profile, and other relevant personal record. Information from third parties including family members and treatment providers may be useful as well. Generally video surveillance is not recommended, as it cannot record internal emotional states. Start interview with open ended questions in order to explore symptoms followed by mental state examination and continues with inquiries to assess specific areas of functioning. The safety of conducting psychiatrist assessments is of prime importance specially if an evaluee is suicidal or homicidal during the disability evaluation.

The next part of the interview should focus on examining the internal consistency of claimants report to correlate requirements of job with his/her impairments. It is important to look for factors related to circumstances outside the work place. This could be done by comparing mood, speech, behavior, and thought processes during the interview to detect malingering and minimize real gain. One clue for psychological gain could be, if the evaluee didn’t show interest in seeking treatment. Additionally when reliability of patient interview and diagnosis is an issue, neuropsychological testing should be performed. For this purpose Wechsler Adult Intelligence Scale-III (WAIS-III) and Minnesota Multiphasic Personality Inventory (MMPI-2) are sometimes used for conducting cognitive testing in disability evaluations (Melton et al., 2007).

Psychiatrists are often asked to report whether an evaluee’s signs and symptoms limit or restrict a person’s ability to perform occupational functions. It is better to comment on Multiaxial diagnosis, including GAF score and use current Diagnostic and Statistical Manual of Mental Disorders (DSM) categories for making Diagnosis. Define impairments in work function; recommend treatment if any and possible course the illness might take.

Psychiatrists are often asked to justify their reports. They should be able to give evidence and support their evaluations based on their judgment, to stand a trial whenever called upon. Americans with Disabilities Act Amendments Act (ADAAA) legislation therefore recommends complete and careful examination even if the evaluee is asymptomatic. The clinician who performs disability evaluation is taken in lieu to the standards of a forensic psychiatrist if questions arise. Having a psychiatric disorder does not automatically denotes impairment or qualifies for a disability.

Honesty is another subjective issue (Appelbaum, 1990, 1997; Griffith, 1998; Shuman and Greenberg, 1998; Candilis et al., 2007) that needs to be addressed, in order to avoid bias. If litigation occurs after evaluation is conducted, then a psychiatrist is not obliged to release information that did not become public in order to avoid legal liabilities (Weinstock and Garrick, 1995; Appelbaum, 2001; Binder, 2002; Gold and Davidson, 2007).

Although no fixed pattern (Group for the Advancement of Psychiatry, 1991; Allnutt and Chaplow, 2000; Silva et al., 2003) has been approved, the report should be prepared according to the format of a standard forensic psychiatric report unless specified. Following AAPL’s guidelines a psychiatrist should be able to perform a well formulated and accurate disability evaluation (Sugarman, 1996). Honest disagreement can be encountered which must be respected. If somehow a psychiatrist is unable to reach a decision and has doubts about certainty, he/she can notify the referral source. Disability decision is an administrative and a legally based process. It does not solely depend on a psychiatrists evaluation report, but is given a prime importance when the court makes a decision. Table 2 (Douglas et al., 2007) summarizes what a typical report should include.

## Conflict of Interest Statement

The authors declare that the research was conducted in the absence of any commercial or financial relationships that could be construed as a potential conflict of interest.
